# Structural characterization of HCoV-229E and H163A main protease (M^pro^) interactions with the inhibitor narlaprevir

**DOI:** 10.1016/j.bbrep.2026.102821

**Published:** 2026-10-04

**Authors:** Gaoyang Yuan, Pei Zeng, Xuelan Zhou, Li Guo, Wenwen Li, Jiahua Cui, Jian Li

**Affiliations:** a Jiangxi Province Key Laboratory of Pharmacology of Traditional Chinese Medicine, School of Pharmacy, Gannan Medical University, Ganzhou, 341000, China; b Jiangxi Jmerry Biopharmaceutical Co., Ltd., Ganzhou, 341000, China

**Keywords:** HCoV-229E, H163A, Main protease, Inhibitor, Narlaprevir

## Abstract

Narlaprevir, originally developed as a clinical-stage inhibitor of the hepatitis C virus (HCV) NS3/4A protease, has recently demonstrated potent inhibitory activity against the SARS-CoV-2 main protease (M^pro^), with its co-crystal structure already resolved. To elucidate the mechanistic basis of its cross-genus inhibition and drug resistance profiles, we determined the crystal structures of narlaprevir in complex with HCoV-229E M^pro^ and the SARS-CoV-2 M^pro^(H163A) mutant at resolutions of 2.01 Å and 2.14 Å, respectively. By integrating these crystallographic data with molecular dynamics (MD) simulations and Molecular Mechanics/Poisson-Boltzmann Surface Area (MM/PBSA) free energy calculations, we systematically compared narlaprevir against clinically approved antivirals, including bofutrelvir, PF-07304814, and pomotrelvir. Structural analysis revealed that narlaprevir forms a conserved covalent C–S bond with the catalytic cysteine residue across all investigated complexes. Thermodynamic evaluations indicated that the SARS-CoV-2 WT-M^pro^–narlaprevir complex exhibits the most negative total binding free energy (−66.29 kcal/mol), suggesting a marginally stronger theoretical binding affinity compared to the 229E–narlaprevir (−64.91 kcal/mol) and H163A–narlaprevir (−64.14 kcal/mol) complexes. Notably, distinct from the approved drugs, narlaprevir exclusively occupies protomer A when bound to both HCoV-229E and H163A M^pro^. Collectively, these findings establish narlaprevir as a highly promising lead compound with notable broad-spectrum potential. While its overall efficacy parallels that of clinically approved agents, it exhibits distinct binding characteristics. These insights provide a critical structural foundation for the rational design of next-generation antiviral strategies capable of mitigating threats from both circulating and emerging coronaviruses.

## Highlights

• First structural characterization of narlaprevir bound to HCoV-229E M^pro^ and the SARS-CoV-2 M^pro^(H163A) mutant.
• Integrated multi-scale approach combining X-ray crystallography, MD simulations, and MM/PBSA calculations to dissect binding mechanisms.
• Narlaprevir exhibits striking conformational conservation across distinct coronavirus lineages despite subtle interaction variations.
• Reveals a structurally conserved inhibition mechanism driven by the high homology of the core catalytic cleft.
• Provides a robust structural template for designing next-generation pan-coronavirus antivirals against emerging threats.

## Graphical abstract

**Figure:**
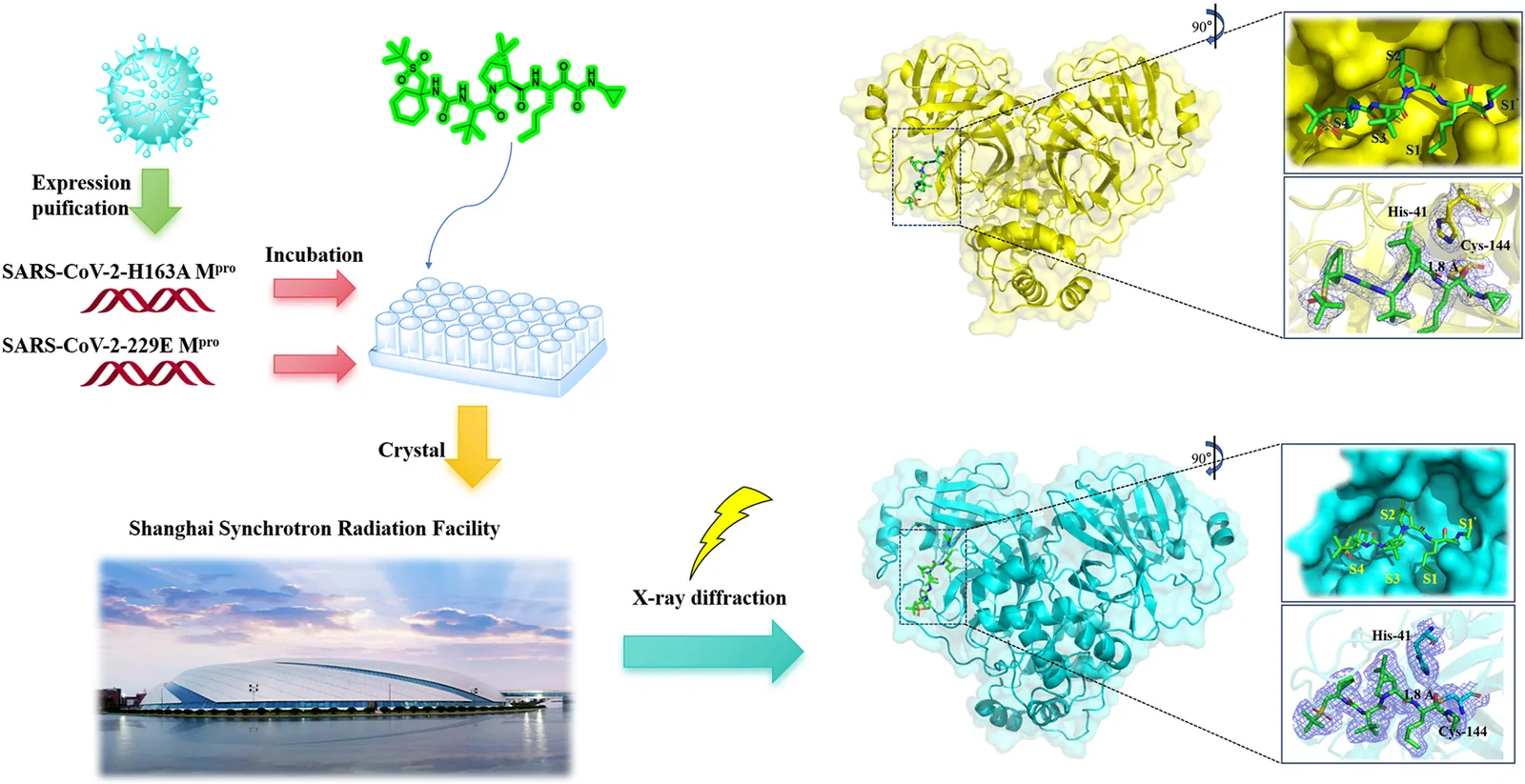

## 1 Introduction

Although the most acute phase of the coronavirus pandemic has subsided [1], history offers a sobering reminder: novel coronaviruses have repeatedly spilled over into human populations and are likely to do so again [2,3]. Far from disappearing, SARS-CoV-2 appears poised to become an endemic fixture in human communities for years to come [4,5]. As it circulates globally, the virus continues to evolve under immune and therapeutic pressures. While variants with enhanced transmissibility or antibody escape capabilities have already emerged [6], the next evolutionary step could be even more alarming: the emergence of strains resistant to current antivirals. This looming threat underscores an urgent need for next-generation therapeutics capable of targeting a broad spectrum of coronaviruses, rather than focusing solely on the currently dominant strain [7].

Central to the viral replication machinery is the main protease (M^pro^), also known as 3C-like protease (3CL^pro^), a cysteine protease essential for the coronavirus life cycle. M^pro^ cleaves the viral polyproteins pp1a and pp1ab at 11 highly conserved sites, releasing nonstructural proteins required for transcription, genome replication, and virion assembly [8]. The compelling nature of M^pro^ as a drug target stems from the absence of functional orthologs in humans [9], allowing for selective viral inhibition with minimal risk of off-target effects—a rare advantage in antiviral development [10]. Although numerous M^pro^ inhibitors have shown promise in preclinical studies over the past few years [[11], [12], [13], [14]], many candidates have faltered in clinical translation due to insufficient potency, unexpected cytotoxicity, or unfavorable pharmacokinetic profiles [15].

One promising candidate is narlaprevir, originally developed by Kyowa Hakko Kirin (Japan) as a potent inhibitor of hepatitis C virus (HCV) NS3/4A protease [16]. Having advanced to clinical trials for chronic hepatitis C, narlaprevir has recently re-emerged as a potent inhibitor of SARS-CoV-2 M^pro^.

HCoV-229E, an α-coronavirus [17], exhibits significant intergeneric divergence from the β-coronavirus SARS-CoV-2. Consequently, investigating the HCoV-229E M^pro^ is of paramount importance for elucidating the cross-genus inhibitory mechanisms of therapeutic compounds. The H163A mutant, generated by substituting the histidine at position 163 with alanine in the wild-type SARS-CoV-2 M^pro^, serves as a critical model system in structural and functional studies of this protease. Previous studies [18] have demonstrated that the side chain of His163 is essential for inhibitor binding; its replacement with alanine leads to a marked reduction in the computationally predicted binding affinities of various established inhibitors. Therefore, this mutant is of significant value for evaluating alterations in both the binding modes and inhibitory potencies of candidate compounds.

Although the co-crystal structure of the SARS-CoV-2 M^pro^–narlaprevir complex has been resolved [19], elucidating its precise target engagement mechanism, the structural basis underlying the molecular interactions between narlaprevir and both the α-coronavirus HCoV-229E M^pro^ and the H163A mutant remains uncharacterized. This critical structural gap severely limits our comprehensive understanding of narlaprevir's cross-genus inhibitory mechanisms and drug resistance profiles, thereby posing a significant impediment to the rational design of broad-spectrum antivirals capable of addressing viral evolution and cross-genus infections [20].

Here in, we report the high-resolution (2.01 Å and 2.14 Å) three-dimensional structures of HCoV-229E M^pro^ and H163A M^pro^ in complex with narlaprevir. Our structures reveal the precise molecular contacts enabling narlaprevir to bind both an α-coronaviral protease and the H163A variant, highlighting conserved structural motifs shared across α- and β-coronaviruses that underpin broad-spectrum inhibition. Furthermore, we systematically compared the binding mode of narlaprevir with HCoV-229E M^pro^ against that observed in the H163A M^pro^–narlaprevir complex to elucidate key structural and mechanistic distinctions. By performing comparative molecular dynamics (MD) simulations of the wild-type 229E, SARS-CoV-2-H163A M^pro^ and wild-type SARS-CoV-2 M^pro^ –narlaprevir mutant complexes, combined with our crystallographic data, we provide a mechanistic understanding of the residue-level determinants governing high-affinity inhibition. Collectively, these results establish a robust foundation for the structure-guided design of next-generation antivirals—agents that are not only more potent but also resilient against the inevitable evolution of future coronavirus variants.

## 2 Materials and methods

### 2.1 Expression and purification of HCoV-229E M^pro^ and H163 M^pro^

The gene encoding the M^pro^ of human coronavirus 229E (HCoV-229E) [21]and H163A M^pro^ was commercially generated by gene synthesis using *E. coli*-optimized codons and subcloned into the pET-28a plasmid, resulting in an N-terminal hexahistidine (6×His) tag fusion. The recombinant plasmid was introduced into chemically competent *E. coli* Rosetta (DE3) cells to enable protein expression. Expression and purification were carried out following established protocols previously described by our group [22]. To ensure native N-terminal integrity, the 6×His tag was subsequently cleaved using tobacco etch virus (TEV) protease, and the released M^pro^ was subsequently purified to homogeneity by gel filtration chromatography.

### 2.2 Crystallization of HCoV-229E M^pro^ and H163A M^pro^ in complex with narlaprevir

Following the team's previously established protocols [23], narlaprevir was incubated with the purified human coronavirus 229E M^pro^ and H163A M^pro^ at a concentration of 10 mg mL^−1^, at a molar ratio of 1:3, at 0 °C for over 30 min to form a stable complex. Crystallization trials were carried out at 20 °C, yielding high-quality crystals under the optimized condition: 10% (v/v) 2-propanol, 0.1 M HEPES buffer (pH 6.5), and 20% (w/v) polyethylene glycol 3350 (PEG 3350).

### 2.3 Data collection, structure determination, and refinement

Prior to flash-cooling in liquid nitrogen, the crystals underwent brief cryoprotection by soaking in a reservoir solution containing 20% (v/v) glycerol. X-ray diffraction data were subsequently collected using the same approach as in our previous studies [24]: utilizing beamline BL02U1 at the Shanghai Synchrotron Radiation Facility (SSRF), all datasets were collected at 100 K. Subsequent automatic processing of diffraction images was conducted using the Aquarium pipeline [25], with detailed collection and processing statistics listed in Table 1. The structure of the HCoV-229E M^pro^ in complex with narlaprevir was solved by molecular replacement with Phaser [26], using a homologous coronavirus M^pro^ structure as the search model. Iterative cycles of refinement were performed in PHENIX [27] employing maximum-likelihood target functions, while manual model building and adjustment were carried out in Coot. The final refined model was evaluated for stereochemical accuracy with MolProbity [28]. Key refinement metrics for the HCoV-229E M^pro^–narlaprevir and H163A M^pro^-narlaprevir complex are summarized in Table 1. All structural illustrations, including binding-site depictions and conformational overlays, were produced using PyMOL.

**Table 1:** Statistics for data processing and model refinement of M^pro^-Narlaprevir (cocrystal).

Parameter	HCoV-229E M^pro^–Narlaprevir	SARS-CoV M^pro^ H163A-Narlaprevir
**PDB code**	9XXJ	24DE
**Date collection**		
Synchrotron	SSRF	SSRF
Beam line	BL02U1	BL02U1
Wavelength (Å)	0.97918	0.97918
Space group	P 1 21 1	P 21 21 21
a, b, c (Å)	53.88, 75.48, 77.13	68.2103.07 104.11
α, β, γ (°)	90.00, 90.98, 90.00	90.0 90.0 90.0
Total reflections	246670	499388
Unique reflection	40954	41193
Resolution (Å)	2.01 (2.06 - 2.01)	2.14 (2.2 - 2.14)
R-merge (%)	9.9(81.7)	22.5(142.0)
Mean I/σ (I)	12.3/2.1	11.0/2.0
Completeness (%)	97.7(94.8)	99.8(99.9)
Redundancy	6.0(4.1)	12.1(11.4)
**Refinement**		
Resolution (Å)	44.52- 2.01	49.91-2.14
Rwork/Rfree(%)	20.28/24.30	22.51/27.84
Atoms	4823	4715
Mean temperature factor (Å2)	41.7	41.7
Bond lengths (Å)	0.007	0.01
Bond angles (°)	0.915	1.02
Ramachandran plot (%)		
Preferred	96.45	96.28
Allowed	3.55	3.72
outliers	0	0

Values in parentheses are for the highest-resolution shell.

### 2.4 Data availability

Data for the HCoV-229E M^pro^–narlaprevir and H163A M^pro^–Narlaprevir complex, including atomic coordinates and structure factors, have been archived in the Protein Data Bank under PDB ID 9XXJ and PDB ID 24DE.

### 2.5 Molecular dynamics (MD) simulations and MM/PBSA binding free energy calculations

In this study, 100-ns molecular dynamics (MD) simulations were performed on the protein–ligand complexes obtained from molecular docking using the GROMACS v2022.03 package. The detailed simulation protocols and parameter settings are described as follows:

(1) System Preparation and Topology Generation: The protein structure was converted into a.gro coordinate file, and the corresponding topology (.top) and force field parameter files were generated using the pdb2gmx module in GROMACS, with the AMBER99SB-ILDN force field selected. For the small molecule, the Generalized Amber Force Field (GAFF) parameters were assigned using AmberTools 22. Additionally, Gaussian 16W was employed for hydrogen addition and the calculation of Restrained Electrostatic Potential (RESP) charges, which were subsequently incorporated into the MD system topology.
(2) Solvation and Neutralization: The protein–ligand complex was solvated using the Three-point Transferable Intermolecular Potential (TIP3P) water model. A minimum distance of 1.2 nm was maintained between the protein atoms and the edges of the cubic simulation box. Appropriate amounts of Na^+^ ions were added to neutralize the overall charge of the simulation system.
(3) Energy Minimization: To eliminate steric clashes and obtain a stable system, energy minimization (EM) was conducted using the steepest descent algorithm.
(4) Interaction Parameters: A cutoff distance of 1.0 nm was applied for short-range van der Waals and electrostatic interactions. Long-range electrostatic interactions were evaluated using the Particle Mesh Ewald (PME) method. All bonds involving hydrogen atoms were constrained using the LINCS algorithm.
(5) Thermostat and Barostat: During the MD simulations, the system temperature was regulated using the V-rescale thermostat, and the pressure was maintained at 1 bar using the Berendsen barostat.
(6) Equilibration: Prior to the production run, the system underwent a 100-ps pre-equilibration to optimize the initial conformation of the protein in the solvent environment. This was followed by a 100-ps equilibration in the canonical (NVT) ensemble to heat the system to the target temperature, and subsequently by a 100-ps equilibration in the isothermal-isobaric (NPT) ensemble to stabilize the system pressure.
(7) Production MD: Following energy minimization and equilibration, a 100-ns production MD simulation was performed for each protein–ligand complex under periodic boundary conditions (PBC) in the NPT ensemble. The system temperature was maintained at 300 K, and the pressure was kept at 1 bar. An integration time step of 2 fs was used, and trajectory frames (including coordinates, velocities, and energies) were saved every 10 ps for subsequent analyses [29,30].

Subsequently, the binding stability of these protein–ligand complexes was quantified by analyzing the equilibrated final 20-ns segments of the MD trajectories with the gmx_MMPBSA tool (built upon MMPBSA.py v16.0) [31]. The binding free energy (Δ_Gbind_) of the protein–ligand complexes was calculated using the MM/PBSA (Molecular Mechanics-Poisson-Boltzmann Surface Area) methodology [32]. The MM/PBSA framework decomposes Δ_Gbind_ into two major components: the total binding free energy is decomposed into the molecular mechanics energy in vacuum (Δ_Ggas_) and the solvation free energy (Δ_Gsolv_). Specifically,Δ_Ggas_ comprises the van der Waals (Δ_EvdW_) and electrostatic (Δ_Eelec_) interaction terms. Meanwhile,ΔG_solv_ is partitioned into a polar component (Δ_Gpolar_, estimated via Δ_EGB_) and a nonpolar component (Δ_Gnonpolar_, approximated by Δ_Esurf_). Calculations of the binding free energy (Δ_Gbind_) were performed using the following equations:

$$\Delta G_{\text{bind}}=\Delta G_{\text{gas}}+\Delta G_{\text{solv}}=\left(\Delta E_{\text{vdW}}+\Delta E_{\text{elec}}\right)+\left(\Delta G_{\text{polar}}+\Delta G_{\text{nonpolar}}\right)$$

The binding free energy (ΔGbind) serves as a pivotal parameter for assessing the binding affinity and structural stability of protein–ligand complexes, with lower values typically indicating stronger interactions and higher binding stability.

## 3 Results

### 3.1 Crystal structure of HCoV-229E M^pro^ in complex with narlaprevir

To elucidate the mechanism underlying narlaprevir's inhibition of HCoV-229E main M^pro^, We elucidated the three-dimensional structure of the M^pro^–narlaprevir complex at 2.01 Å resolution (Table 1). The enzyme adopts a canonical homodimeric architecture comprising three domains connected by an interdomain loop. Narlaprevir binds within the catalytic cleft between domains I and II, occupying the S1, S2, S4, and S1′ subsites in an extended conformation (Fig. 1A and B). The catalytic center features the conserved Cys144–His41 dyad (Fig. 1 C). Unambiguous electron density reveals a covalent thioether bond (1.8 Å) between the electrophilic warhead of narlaprevir and Cys144, confirming an irreversible inhibition mechanism consistent with the SARS-CoV-2 complex.

**Fig. 1:**
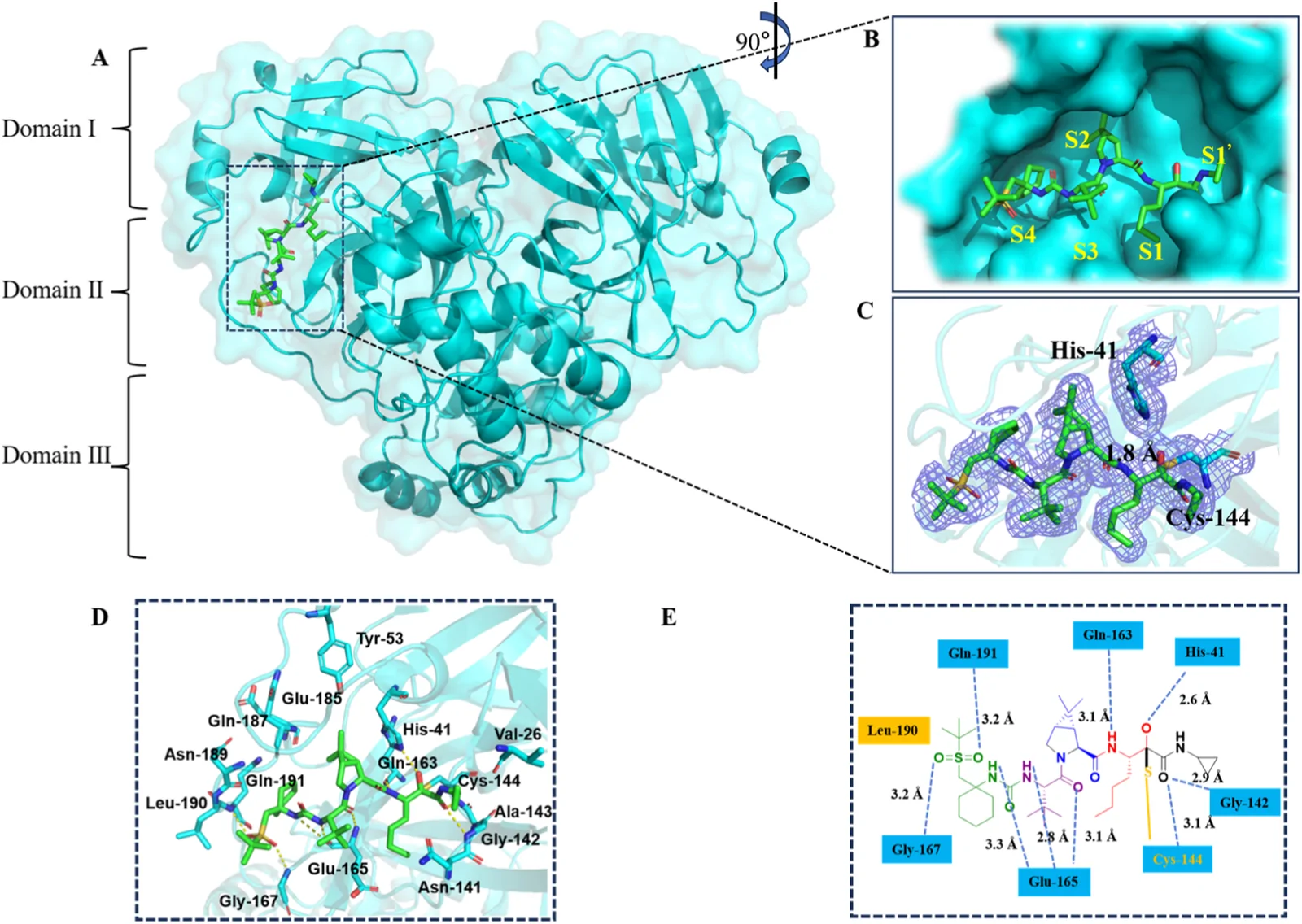
Atomic-resolution structure of HCoV-229E M^pro^ co-crystallized with narlaprevir. (A) Overall structure of the M^pro^ dimer showing domains I–III and the Narlaprevir binding site (dashed box). Narlaprevir is shown as sticks (C: light green, O: dark red, N: light blue, F: pale cyan). (B) Close-up of the binding cavity, with Narlaprevir occupying subsites S1, S2, and S4. (C) 2Fo–Fc electron density map (purple mesh) confirming the Cys144–His41 catalytic dyad and the thioether covalent bond between Cys144 and Narlaprevir (D) Molecular interactions within 3.5 Å; residues (e.g., Leu190). are shown as light blue sticks, with hydrogen bonds indicated by yellow dashed lines. (E) 2D interaction diagram highlighting hydrogen-bonding (blue background) and hydrophobic (yellow background) residues, with interactions denoted by blue dashed and yellow solid lines, respectively.

Analysis of the binding interface (Fig. 1 D) uncovers a tight interaction network: the P1 n-butyl group embeds into the hydrophobic S1 pocket; the P1′ cyclopropylamide moiety forms dual hydrogen bonds with His41, Gly142, and Cys144 within the S1′ subsite; the P2 dimethylcyclopropyl group engages in hydrophobic contacts with Val26, Ala142, and Leu190; the P3 backbone establishes reciprocal hydrogen bonds with Glu165; the P4 cyclohexyl ring inserts into the S4 pocket, hydrogen-bonding to Glu165; and the tert-butyl sulfate oxygens form hydrogen bonds with the backbones of Gly167 and Gly191. Collectively, narlaprevir achieves high-affinity, potent inhibition of HCoV-229E M^pro^ by covalently anchoring to Cys144 and establishing an extensive network of hydrophobic interactions and hydrogen bonds established with pivotal residues located in the catalytic pocket (Fig. 1 E).

### 3.2 Crystal structure of H163 M^pro^ in complex with narlaprevir

Employing an identical crystallization and data collection strategy, we determined the crystal structure of the H163A M^pro^–narlaprevir complex at 2.14 Å resolution (Table 1). The overall architecture of narlaprevir-bound H163A M^pro^ closely resembles that of HCoV-229E M^pro^ (Fig. 2A and B), retaining the conserved Cys145–His41 catalytic dyad (Fig. 2 C) and forming an equivalent 1.8 Å covalent thioether linkage between the electrophilic warhead carbon of narlaprevir and the thiol sulfur of Cys145 (Fig. 2 C).

**Fig. 2:**
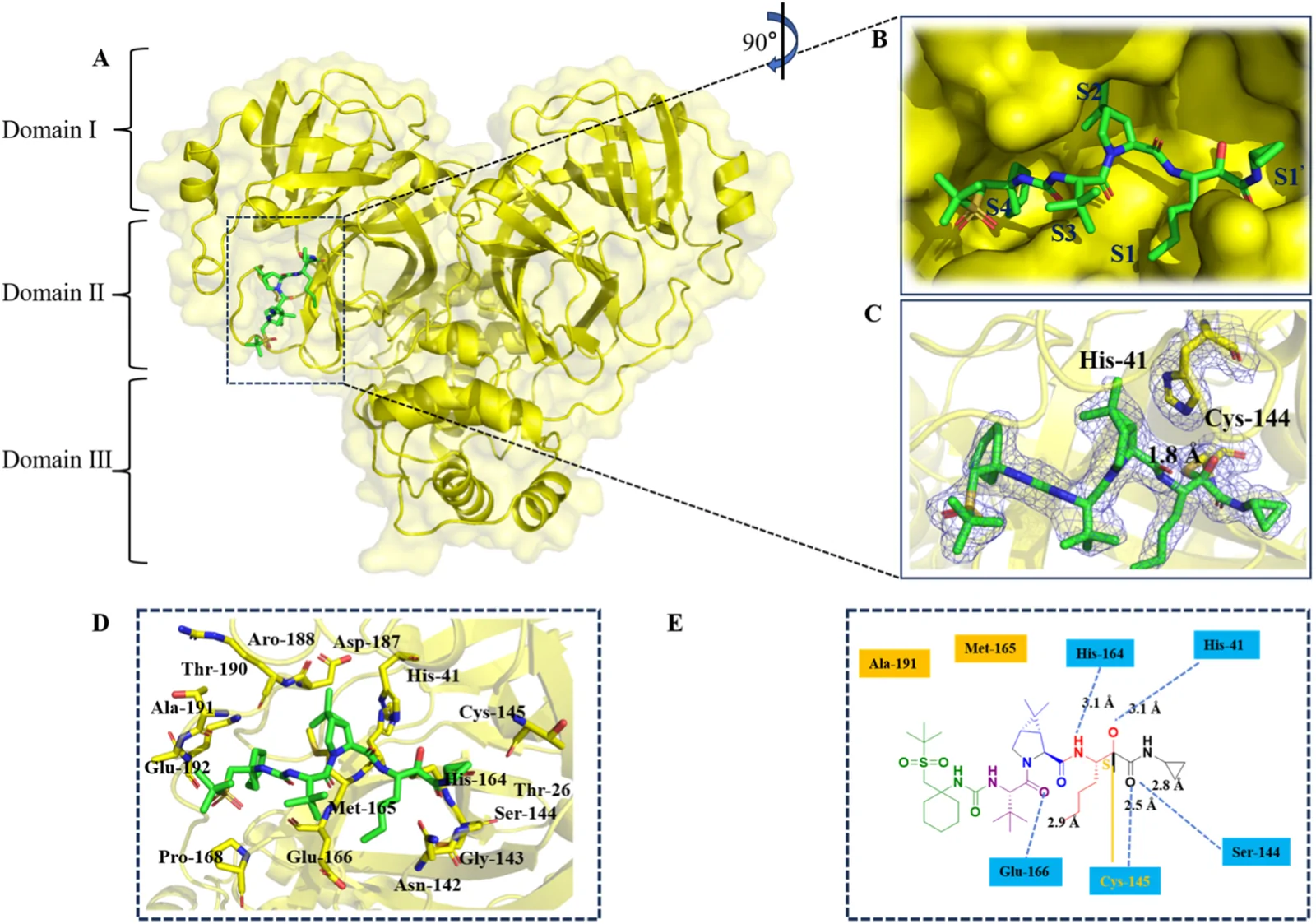
Atomic-resolution structure of H163A M^pro^ co-crystallized with narlaprevir. (A) M^pro^ homodimer structure showing domains I–III and the Narlaprevir binding site (dashed box). Narlaprevir is shown as sticks (C: light green, O: dark red, N: light blue, F: pale cyan). (B) Close-up of the binding cavity with Narlaprevir occupying subsites S1, S2, and S4. (C) 2Fo–Fc electron density map (purple mesh) confirming the Cys145–His41 catalytic dyad and the thioether covalent bond; Cys145/His41 are yellow sticks, Narlaprevir is element-colored. (D) Interactions within 3.5 Å; surrounding residues (e.g., Ala191) are yellow sticks, with hydrogen bonds shown as yellow dashed lines. (E) 2D interaction diagram highlighting hydrogen-bonding (blue background) and hydrophobic (yellow background) residues; interactions are denoted by blue dashed lines, and the covalent bond by a yellow solid line.

We analyzed the interactions between narlaprevir and amino acid residues within a 3.5 Å radius of the H163A M^pro^ binding site. In contrast to HCoV-229E M^pro^, H163A M^pro^ engages in a reduced number of hydrogen-bonding interactions with narlaprevir, with conserved contacts largely localized to the right side of the binding interface (Fig. 2 E). Specifically, the P1′ cyclopropylamide moiety occupies the adjacent S1′ subsite, wherein its carbonyl oxygen functions as a hydrogen bond acceptor from the Nε–H of His41, while its amide oxygen serves as a donor to the backbone amides of Ser144 and Cys145. Additionally, His164 near the S1 pocket acts as a hydrogen bond acceptor, forming a hydrogen bond with the amide hydrogen adjacent to the DMCP on the main chain, while the P2 DMCP establishes hydrophobic interactions with Met165. In the central region (Fig. 2 E), Glu166 at the P3 position establishes hydrogen bonds involving the main-chain carbonyl oxygen and amide nitrogen.

Collectively, narlaprevir achieves high-affinity inhibition through covalent anchoring to Cys145, complemented by an extensive hydrogen-bonding network involving His41, Ser144, Cys145, His164, and Glu166, along with hydrophobic contacts with Ala191 and Met165 (Fig. 1 E). This comprehensive engagement of the active site underpins the potent inhibitory activity of narlaprevir against H163A M^pro^.

### 3.3 Comparative structural analysis of HCoV-229E M^pro^–Narlaprevir and H163A M^pro^–Narlaprevir complexes revealing key mechanistic insights

To gain mechanistic insights into pan-inhibitory activity of narlaprevir against coronaviral M^pro^s, we performed a comparative structural analysis of its binding mode in HCoV-229E M^pro^ versus H163A M^pro^. As shown in the superimposed structures (Fig. 3 A), the overall folds of both proteases adopt highly similar conformations upon covalent engagement with narlaprevir, reflecting a conserved architecture of the catalytic domain across these evolutionarily related viruses [33].

**Fig. 3:**
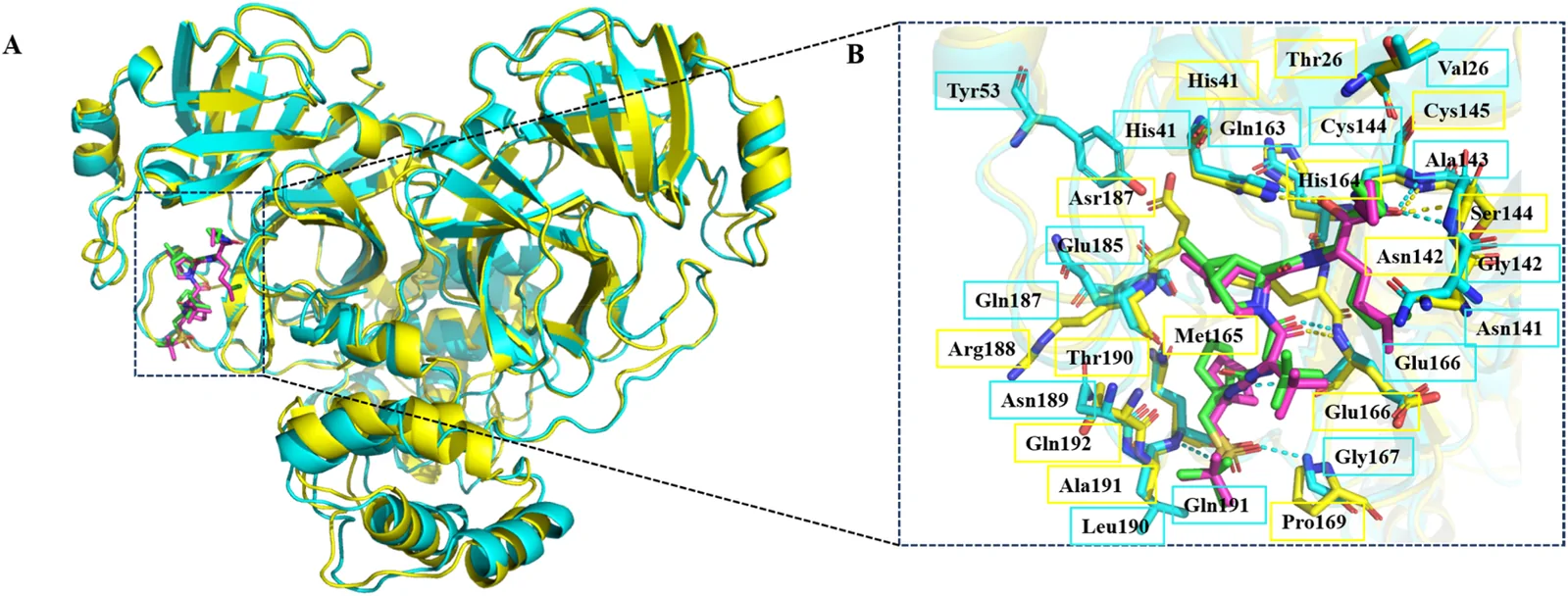
Structural comparison of HCoV-229E M^pro^ and H163A M^pro^ in complex with narlaprevir. (A) Structural comparison of narlaprevir-bound HCoV-229E M^pro^ and H163A M^pro^ monomers. HCoV-229E M^pro^–narlaprevir complex is shown in blue, and H163A M^pro^–narlaprevir complex is shown in yellow. (B) Zoomed-in perspective illustrating the specific interactions (cutoff: 3.5 Å) within the HCoV-229E M^pro^–narlaprevir and H163A M^pro^–narlaprevir complexes. Narlaprevir is rendered as sticks. H-bonds are highlighted by blue dashed lines for the wild-type enzyme and yellow dashed lines for the H163A mutant.

To dissect the molecular determinants underlying inhibitor recognition, we further examined the atomic-level interactions within a 3.5 Å radius of narlaprevir in each complex (Fig. 2 B). Structural alignment reveals that the core substrate-binding cleft—particularly the S1 and S4 subsites—is largely preserved between HCoV-229E and H163A M^pro^, consistent with their shared chymotrypsin-like fold and catalytic dyad (His41-Cys144/145) [21]. In both structures, narlaprevir forms the same covalent thioether linkage with the catalytic cysteine–Cys144 in HCoV-229E and Cys145 in H163A—with the C–S bond measuring a consistent 1.8 Å. Furthermore, in both structures, His41, Cys144, Gln163, and Glu165 all establish hydrogen-bonding interactions with narlaprevir. This uniform geometry strongly supports the formation of a stable covalent adduct in both viral proteases.

Although the covalent linkage to the catalytic cysteine is conserved, the surrounding non-covalent interactions exhibit substantial divergence. In the HCoV-229E complex, a markedly greater number of intermolecular forces contribute to structural stabilization, with residues including Val26, Gly142, Gly167, and Gln191 establishing contacts with narlaprevir through hydrogen bonding or van der Waals forces (Fig. 3 B). By contrast, their structural equivalents in H163A M^pro^ reside beyond the typical 3.5 Å cutoff for direct interaction and appear inert in binding. The reverse is also true: H163A M^pro^ leverages Ser144, Met165, and Ala191 to engage the inhibitor via polar and hydrophobic contacts, respectively, yet HCoV-229E lacks residues at these positions that can fulfill comparable roles. These asymmetries underscore how sequence variations can reshape the fine details of inhibitor recognition despite an overall conserved scaffold.

### 3.4 Molecular dynamics analysis

To further elucidate the mechanistic basis underlying the pan-inhibitory activity of narlaprevir against coronavirus M^pros^, we performed molecular dynamics (MD) simulations on three protein–ligand complexes: HCoV-229E M^pro^, SARS-CoV-2 M^pro^ H163A mutant, and SARS-CoV-2 M^pro^ wild-type (WT). As illustrated in (Fig. 4 A), all three systems underwent rapid conformational adjustments during the initial phase of the simulations, subsequently entering a relatively stable plateau [34]. The root mean square deviation (RMSD) of the 229E–narlaprevir complex increased to approximately 0.30–0.40 nm after 20 ns, exhibiting the most pronounced fluctuations among the three systems. This indicates substantial global conformational adaptation of the protein upon ligand binding. In contrast, the RMSD of the H163A–narlaprevir complex was predominantly maintained at ∼0.20–0.26 nm, representing the minimal overall structural deviation across the systems. The WT–narlaprevir complex stabilized at ∼0.22–0.29 nm, displaying an intermediate level of global stability. Collectively, the H163A and WT systems exhibited relatively minor global structural shifts. Although the 229E system experienced more pronounced conformational rearrangements, it ultimately achieved a stable plateau in the latter half of the simulation without continuous structural drift.

**Fig. 4:**
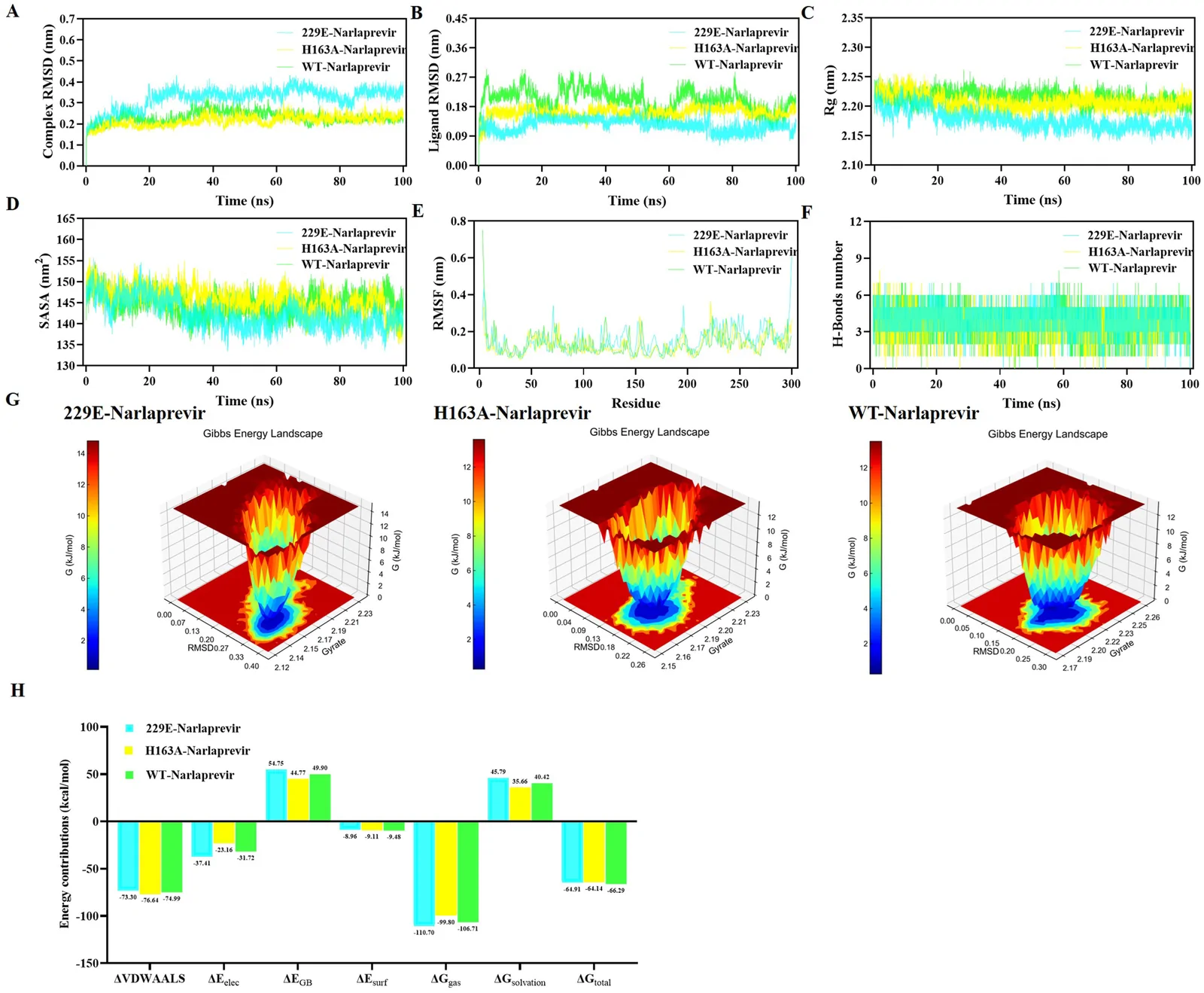
Analysis of 100-ns molecular dynamics (MD) simulations of the complexes. (A) Root mean square deviation (RMSD) profiles of the complexes. (B) RMSD trajectories of the ligand. (C) Radius of gyration (Rg) profiles of the protein. (D) Solvent-accessible surface area (SASA) profiles of the protein. (E) Root mean square fluctuation (RMSF) profiles of the protein. (F) Time evolution of intermolecular hydrogen bonds within the complexes. (G) Free energy landscape (FEL) of the complexes.

Furthermore, ligand RMSD was evaluated to assess the positional stability of the inhibitor within the binding pocket. As shown in (Fig. 4 B), the ligand RMSD in the 229E system was predominantly maintained at ∼0.10–0.16 nm, representing the lowest value among the three. In the H163A system, the ligand RMSD was mainly distributed within the range of ∼0.14–0.18 nm. Conversely, the WT system exhibited relatively higher ligand RMSD values, largely falling between ∼0.18 and 0.24 nm, accompanied by several transient spikes approaching 0.27–0.30 nm. These findings suggest that despite the relatively large global conformational changes observed in the 229E protein, narlaprevir exhibits the highest positional stability within its binding pocket. This implies that the protein likely undergoes adaptive conformational adjustments to further encapsulate or secure the ligand. In contrast, the ligand in the WT system underwent more pronounced local pose adjustments.

The radius of gyration (Rg) serves as a critical metric for evaluating the structural compactness of proteins [35]. Generally, a structurally stable protein exhibits greater compactness, which corresponds to a lower Rg value. As illustrated in (Fig. 4 C), the Rg values across all three systems were consistently maintained within the range of ∼2.15–2.24 nm, without any sustained increase, indicating that no significant unfolding events occurred during the simulations. Notably, the Rg of the 229E system gradually decreased from an initial value of ∼2.20 nm to ∼2.16–2.18 nm, suggesting a progressive compaction of the overall protein structure. In contrast, the H163A and WT systems stabilized predominantly at ∼2.20–2.22 nm and ∼2.21–2.23 nm, respectively, exhibiting minimal fluctuations in structural compactness. Collectively, these results demonstrate that all three systems maintained robust global folding stability throughout the simulations, with the 229E system displaying a more pronounced tendency toward conformational compaction.

Solvent-accessible surface area (SASA) is a widely used metric for evaluating the extent of solvent exposure of hydrophobic and hydrophilic residues, providing insights into the average surface area of amino acids accessible to the solvent and the overall compactness of the system [36]. As shown in (Fig. 4 D), the SASA values across all three systems were predominantly distributed within the range of ∼135–155 nm^2^, without any anomalous increases throughout the simulations. Notably, the SASA of the 229E system exhibited a gradual decline, stabilizing at ∼138–143 nm^2^ during the latter half of the trajectory. This downward trend is highly consistent with the decrease in its Rg, suggesting that surface hydrophobic regions or specific residues progressively collapsed inward, thereby driving the system toward a more compact state. In contrast, the SASA values for the H163A and WT systems were largely maintained between ∼143 and 149 nm^2^, indicating a relatively stable degree of solvent exposure. Taken together, the combined Rg and SASA analyses suggest that the elevated global RMSD observed in the 229E system is more likely attributable to stable conformational rearrangements or structural compaction, rather than protein unfolding.

To investigate the impact of ligand binding on the flexibility of amino acid residues, we calculated the root mean square fluctuation (RMSF) profiles for the entire simulation trajectories. As illustrated in (Fig. 4E), the RMSF values for the majority of residues across the three systems remained below ∼0.20 nm, indicating robust rigidity within the protein's core structural elements. Elevated fluctuations were predominantly localized in loop regions and the N- and C-termini, which is a characteristic feature of the inherent flexibility at protein termini and surface loops. The 229E system exhibited relatively higher RMSF peaks in specific loop regions and the C-terminus, whereas the WT system displayed pronounced transient fluctuations at the N-terminus. The H163A system showed an overall fluctuation pattern comparable to the other systems. These observations suggest that the H163A mutation did not induce a significant increase in global protein flexibility; rather, its effects are likely confined to the local interaction network in the vicinity of the binding pocket.

To further explore the dynamic evolution of hydrogen bonding within the complexes, we performed a statistical analysis of the intermolecular hydrogen bonds formed between the protein and narlaprevir over the 100 ns MD simulations. As shown in (Fig. 4F), all three systems consistently maintained hydrogen-bonding interactions between the protein and narlaprevir throughout the simulations, with the number of hydrogen bonds predominantly ranging from 2 to 6, and occasionally reaching 7 to 8 at specific time points. Despite transient fluctuations in the instantaneous number of hydrogen bonds, none of the three systems experienced a prolonged, complete loss of these interactions. This indicates that narlaprevir remained persistently anchored within the binding pocket throughout the entire simulation period.

The free energy landscape (FEL) analysis (Fig. 4G) revealed that all three systems formed distinct funnel-shaped low-free-energy basins, indicating that they gradually converged from their initial states to relatively stable dominant conformational ensembles during the simulations. The low-energy region of the 229E system was slightly elongated along the RMSD axis, consistent with its larger overall RMSD fluctuations and suggesting a more pronounced conformational adaptation process. In contrast, the low-energy basin of the H163A system was predominantly localized within a lower RMSD range, reflecting a more concentrated overall conformational distribution. The WT system also exhibited a well-defined primary low-energy basin, which corresponded to a relatively higher radius of gyration (Rg) and moderate RMSD values. Collectively, while all three systems populated dominant low-energy conformations, the 229E system displayed a certain degree of conformational heterogeneity, whereas the H163A system maintained a comparatively focused conformational distribution.

The MM/PBSA results (Fig. 4H) demonstrate that the total binding free energies for narlaprevir across all three protein systems (229E, H163A, and WT) are negative, indicating that the formation of these three complexes is thermodynamically favorable. Among them, the WT–narlaprevir complex exhibits the most negative total binding free energy (−66.29 kcal/mol), suggesting a marginally stronger theoretical binding affinity compared to the 229E–narlaprevir (−64.91 kcal/mol) and H163A–narlaprevir (−64.14 kcal/mol) complexes. The binding across the three systems is primarily driven by van der Waals and electrostatic interactions, whereas the positive polar solvation energy exerts a significant opposing effect. Although the H163A mutation substantially attenuates electrostatic interactions, this penalty is counterbalanced by enhanced van der Waals contacts and a reduced solvation penalty, resulting in a total binding free energy comparable to that of the wild-type 229E system. Collectively, the binding capabilities of the three systems are at a similar level, with the WT system displaying a slight energetic advantage.

### 3.5 Comparison of the structure of narlaprevir with other covalent inhibitors bound to HCoV-229E and H163A-M^pro^

Bofutrelvir, PF-07304814, and pomotrelvir exhibit broad-spectrum inhibitory activities against the M^pros^ of diverse coronaviruses. In previous studies, our team determined and deposited the co-crystal structures of bofutrelvir and PF-07304814 in complex with HCoV-229E-M^pro^, as well as those of bofutrelvir and pomotrelvir bound to the H163A mutant M^pro^. We retrieved these publicly available structures from the RCSB PDB database and conducted a comparative structural analysis with the HCoV-229E-M^pro^–narlaprevir and H163A-M^pro^–narlaprevir complexes resolved in this study (Fig. 5).

**Fig. 5:**
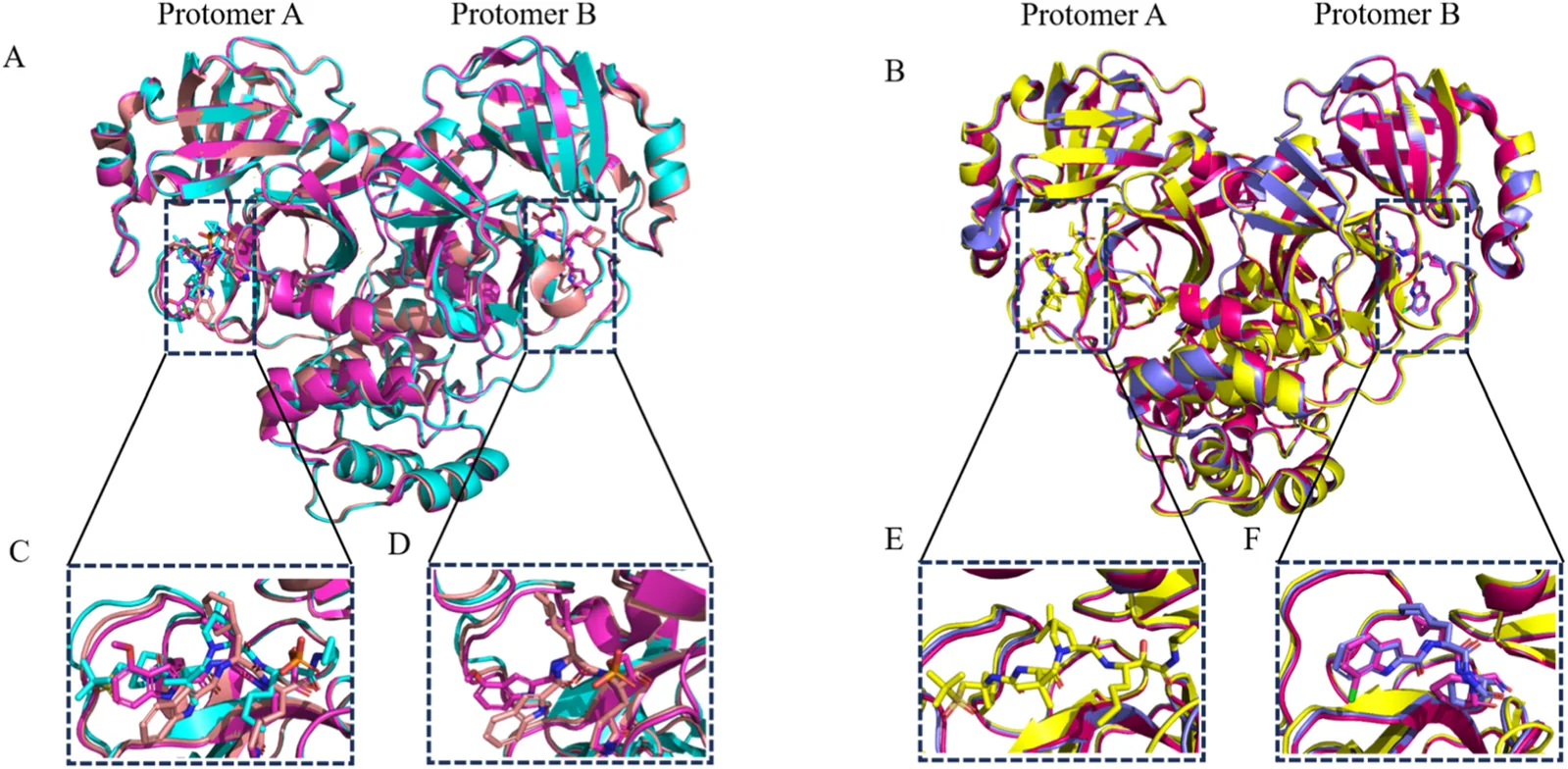
Structural superposition of inhibitor complexes with HCoV-229E and H163A M^pro^. (A) Overall structural superposition of the HCoV-229E-M^pro^ complexes bound to bofutrelvir(8Y4F), PF-07304814(8IM6), and narlaprevir, colored in pink, magenta, and blue, respectively. (B) Overall structural superposition of the H163A-M^pro^ complexes bound to bofutrelvir(8YWZ), pomotrelvir(9M6T), and narlaprevir, colored in blue-violet, peach, and yellow, respectively.(C) Magnified view of the binding pocket in protomer A of the superimposed HCoV-229E-M^pro^ complexes. (D) Magnified view of the binding pocket in protomer B of the superimposed HCoV-229E-M^pro^ complexes. (E) Magnified view of the binding pocket in protomer A of the superimposed H163A-M^pro^ complexes. (F) Magnified view of the binding pocket in protomer B of the superimposed H163A-M^pro^ complexes.

To elucidate the binding discrepancies between narlaprevir and other inhibitors within the HCoV-229E M^pro^, we superimposed the co-crystal structures of HCoV-229E M^pro^ in complex with bofutrelvir, PF-07304814, and narlaprevir for comparative analysis. A shared mechanistic feature among these three inhibitors(Bofutrelvir、PF-07304814 and Narlaprevir) is the formation of a covalent C–S bond with the catalytic cysteine residue of M^pro^. Furthermore, all three inhibitors occupy the S1′, S1, S2, and S4 substrate-binding subsites of the protease. Similar to bofutrelvir and PF-07304814, narlaprevir features a lactam ring that engages in an extensive hydrogen-bonding network—most notably with Glu165—thereby effectively occupying the S1 substrate-binding pocket. Notably, distinct from the other two inhibitors, narlaprevir is exclusively observed in protomer A(Fig. 5 C) of HCoV-229E-M^pro^, whereas bofutrelvir and PF-07304814 are present in both protomers A and B(Fig. 5C and D). Within the S4 subsite, narlaprevir establishes specific hydrogen bonds with Gly167 and Gln191. Collectively, these structural observations indicate that narlaprevir represents a distinct class of covalent inhibitors targeting HCoV-229E-M^pro^, characterized by unique binding features that differentiate it from bofutrelvir and PF-07304814. These findings provide critical structural insights that may facilitate the rational design of next-generation antiviral therapeutics.

To elucidate the binding discrepancies between narlaprevir and other inhibitors within the H163A M^pro^, we superimposed the co-crystal structures of H163A M^pro^ in complex with bofutrelvir, pomotrelvir, and narlaprevir for comparative analysis. Structural analysis revealed that both bofutrelvir and narlaprevir form a covalent C–S bond with the catalytic cysteine residue; in contrast, such a covalent linkage is absent in the H163A M^pro^–pomotrelvir complex.

Furthermore, while all three inhibitors bind exclusively to a single protomer of the H163A M^pro^ dimer, they exhibit distinct protomer preferences: bofutrelvir and pomotrelvir occupy protomer B (Fig. 5 F), whereas narlaprevir is located in protomer A (Fig. 5 E). This selectivity is likely driven by electrostatic complementarity, as protomer B of the H163A mutant displays a more pronounced negative surface potential that favors the binding of bofutrelvir and pomotrelvir, while narlaprevir exhibits an opposing preference [37]. Additionally, all three inhibitors occupy the S1′, S1, S2, and S4 substrate-binding subsites, a conserved binding mode that may underpin their broad-spectrum inhibitory activities.

## 4 Discussion

Narlaprevir, originally developed as a clinical-stage inhibitor of the hepatitis C virus (HCV) NS3/4A protease, has recently demonstrated potent inhibitory activity against the SARS-CoV-2 M^pro^, and its co-crystal structure has been resolved. To elucidate the mechanistic basis underlying its cross-genus inhibition and resistance profiles, we determined the crystal structures of narlaprevir in complex with HCoV-229E M^pro^ and the SARS-CoV-2 M^pro^(H163A) mutant at resolutions of 2.01 Å and 2.14 Å, respectively. In both complexes, the electrophilic warhead of narlaprevir forms a covalent thioether bond with the catalytic cysteine (Cys144 in HCoV-229E; Cys145 in H163A) with a bond length of 1.8 Å, identical to that observed in the SARS-CoV-2 complex. Furthermore, key residues His41, Cys144, Gln163, and Glu165 establish hydrogen-bonding networks with narlaprevir. Notably, distinct variations were observed: while residues Val26, Gly142, Gly167, and Gln191 engage narlaprevir via hydrogen bonds or van der Waals forces in HCoV-229E, their structural equivalents in H163A M^pro^ lie beyond the typical 3.5 Å interaction cutoff. These structural divergences align with the significantly lower RMSD values observed in the H163A–narlaprevir complex. Collectively, this underlying structural conservation likely substantiates narlaprevir's potent inhibition across distinct coronavirus lineages, underscoring its potential as a pan-coronaviral therapeutic scaffold.

We compared the binding modes of narlaprevir with HCoV-229E M^pro^ and H163A M^pro^ against those of the clinically approved drugs bofutrelvir, PF-07304814, and pomotrelvir. In the 229E system, bofutrelvir, PF-07304814, and narlaprevir all form covalent C–S bonds with the catalytic cysteine residue of M^pro^. However, a notable distinction is that narlaprevir is observed exclusively in protomer A of HCoV-229E M^pro^, whereas bofutrelvir and PF-07304814 are present in both protomers A and B. In the H163A system, both bofutrelvir and narlaprevir also form covalent C–S bonds with the catalytic cysteine residue. Although all three inhibitors (bofutrelvir, pomotrelvir, and narlaprevir) bind to only a single protomer of the H163A M^pro^ dimer, they exhibit distinct protomer preferences: bofutrelvir and pomotrelvir occupy protomer B, whereas narlaprevir is localized in protomer A. This selectivity is likely driven by electrostatic complementarity, as protomer B of the H163A mutant displays a more pronounced negative surface potential that favors the binding of bofutrelvir and pomotrelvir, while narlaprevir exhibits an opposing preference. Nevertheless, across both HCoV-229E and H163A M^pro^, all four inhibitors consistently occupy the S1′, S1, S2, and S4 substrate-binding subsites. This conserved binding mode likely underpins their broad-spectrum inhibitory activities.

In conclusion, this study highlights narlaprevir as a highly promising lead compound with notable broad-spectrum potential. While its overall efficacy is comparable to that of clinically approved drugs such as bofutrelvir, PF-07304814, and pomotrelvir, it exhibits distinct and unique characteristics in its binding mode. Collectively, these findings lay a robust foundation for the structure-guided design of next-generation antiviral therapeutics.

## CRediT authorship contribution statement

**Gaoyang Yuan:** Writing – original draft. **Pei Zeng:** Writing – original draft, Visualization. **Xuelan Zhou:** Validation, Investigation. **Li Guo:** Software, Investigation, Formal analysis. **Wenwen Li:** Validation, Investigation. **Jiahua Cui:** Conceptualization. **Jian Li:** Writing – review & editing, Supervision, Investigation.

## Funding

This work was supported by a grant from the 10.13039/501100001809National Natural Science Foundation of China (82360701), Ganzhou city key research and development plan project (GZ2024ZDY043), 10.13039/501100020784Gannan Medical University (QD201910), 10.13039/501100004479Jiangxi Natural Science Foundation (20252BAC250108), Jiangxi “Double Thousand Plan (jxsq2019101064)” and Jiangxi key research and development program (20203BBG73063).

## Declaration of competing interest

This study was not published elsewhere for publication. No conflict of interest exits in the submission of this manuscript, and manuscript is approved by all authors for publication.

## Data Availability

Data will be made available on request.
